# Age-Related and Sex Differences in Visuospatial Working Memory and Its Association with Math Achievement: Insights from Span, Accuracy, and RT in Corsi Block Tapping Test

**DOI:** 10.11621/pir.2025.0205

**Published:** 2025-06-01

**Authors:** Sergey B. Malykh, Yulia V. Kuzmina

**Affiliations:** a Federal Scientific Center for Psychological and Interdisciplinary Research, Moscow, Russia

**Keywords:** visuo-spatial working memory, Corsi Block Tapping Test, sex difference, age difference, math achievement

## Abstract

**Background:**

Visuospatial working memory (VSWM) is critical for academic achievement, particularly in mathematics. The Corsi Block-Tapping Test (CBTT) is one of the most widely used tools to assess VSWM, traditionally relying on span length as the primary performance indicator. However, recent research suggests that additional metrics, such as accuracy and reaction time (RT), may offer complementary insights. Despite this, RT remains underexplored in studies examining VSWM development and its links to academic outcomes such as math performance.

**Objective:**

To investigate age- and sex-related changes in VSWM using CBTT accuracy, span, and RT, and to examine how these metrics relate to math achievement across school grades and sexes.

**Design:**

Data were collected from 2,749 Russian pupils (53% girls), aged 10 to 18 years (M = 13.89, SD = 2.08), enrolled in grades 5 to 11 in two regions (Nizhny Novgorod and Irkutsk). Regression analysis was applied to three indicators of CBTT and math achievement that was measured by average school grades.

**Results:**

CBTT accuracy increased and RT decreased from grades 5 to 8, then both increased post-grade 9, suggesting a developmental shift. Accuracy predicted math grades in grades 5–9 but not later; RT was a stable negative predictor across all grades. Sex did not moderate VSWM–math associations, though girls showed greater RT efficiency in late adolescence.

**Conclusion:**

VSWM develops along non-linear trajectories that differ by metric. Multi-indicator assessment is essential, and school grades should be interpreted with caution as proxies for math ability.

## Introduction

Visuospatial working memory (VSWM), a core component of human cognition, enables people to temporarily store and manipulate visual and spatial information, playing a vital role in tasks such as navigation, problem-solving, and academic learning. One of the most influential frameworks for understanding working memory is Baddeley’s model (Baddeley, 2012), which conceptualizes it as comprising three core components: the phonological loop (for verbal information), the visuospatial sketchpad (for visual and spatial data), and the central executive (for attentional control and coordination). A later revision added the episodic buffer to account for the integration of multimodal information and its interface with long-term memory.

### Age and Gender Differences in VSWM

VSWM develops significantly throughout childhood and adolescence. Longitudinal and cross-sectional research have shown that its capacity, as measured by tasks such as the digit span and the Corsi Block Tapping Test (CBTT), follows a nonlinear developmental trajectory (Ahmed et al., 2022; Tikhomirova et al., 2020a). Typically, VSWM shows rapid growth in early and middle childhood, followed by a plateau during late childhood and late adolescence. For instance, Ahmed et al. (2022) found that forward and backward span performance increased most rapidly during early years and showed a secondary growth spurt in early adolescence, possibly reflecting neurological and educational transitions (Gómez et al., 2018; Kim et al., 2021). Tikhomirova et al. (2020a) highlighted the role of formal schooling over chronological age in explaining VSWM development among Russian students, emphasizing the importance of educational experience in shaping cognitive capacities.

Gender differences in VSWM have been widely studied but remain subject to debate. Some findings suggest that males outperform females in visuospatial tasks, including the CBTT (Voyer et al., 2017; Zilles et al., 2016), while other failed to demonstrate these difference (e.g. Burggraaf et al., 2018). These differences, however, appear to be developmentally dynamic. Some research reports gender differences in early school age (e.g., Leon et al., 2014), while others show that disparities become more pronounced during adolescence (Pauls et al., 2013; Tikhomirova et al., 2020a). Particularly, Tikhomirova et al. (2020a) found differing VSWM growth trajectories between boys and girls during the school years, with boys initially outperforming girls, who later catch up—suggesting convergence by the end of secondary school. However, meta-analysis demonstrated that gender difference increased across age (Voyer et al., 2017).

### VSWM and Mathematics

Many studies have demonstrated that working memory—especially its visuospatial component—is closely linked to academic achievement, particularly in mathematics. While both verbal and visuospatial WM are correlated with school performance, VSWM appears to contribute more specifically to arithmetic skills (Allen et al., 2019; Caviola et al., 2020; Vieira et al., 2021). For example, Vieira et al. (2021) found that VSWM accounted for 38% of the variance in arithmetic performance, whereas no such association was observed for reading. It was also shown that the effect of VSWM varied for different type of math tests. Particularly, VSWM was significantly related to oral and mental arithmetic, but not to spelling or fact retrieval (Allen & Dowker, 2022).

Importantly, this relationship evolves with age. Studies suggest that VSWM is a stronger predictor of math performance in younger children, potentially due to early reliance on spatial representations in math tasks (Allen et al., 2019; Tikhomirova et al., 2020b). Specifically, it was found that VSWM’s predictive value was greatest in primary school, but diminished in later years, particularly when fluid intelligence and processing speed were statistically controlled (Tikhomirova et al., 2020b). In addition, it was shown that the association between working memory and mathematics was stronger in individuals with math difficulties than in those who were typically developing (Peng et al., 2016). This suggests that while VSWM may provide a foundational scaffold for math learning, its relative contribution decreases across development.

Emerging research into gender differences in the relationship between working memory and math achievement reveals nuanced patterns. Some studies showed that gender can moderate association between WM and mathematics (Ganley & Vasilyeva, 2014; Van de Weijer-Bergsma et al., 2021). Particularly, Van de Weijer-Bergsma et al. (2021) found that while both WM modalities were generally predictive of math outcomes, gender moderated these associations. For girls, verbal and visuospatial WM were equally predictive, whereas for boys, verbal WM emerged as the stronger predictor, particularly for problem-solving tasks. Other studies showed that in contrast, the verbal component of working memory was more predictive for girls (Kuzmina et al., 2019). It is possible that the diversity of findings is related to the variety of math tests and instruments for assessing different dimensions of working memory.

### Corsi Block Tapping Test (CBTT)

The Corsi Block Tapping Test (CBTT), originally developed by Philip M. Corsi (1972) in his dissertation “Human memory and the medial temporal region of the brain”, remains one of the most commonly used tools to assess VSWM. In its standard format, the CBTT involves a sequence of spatial locations tapped by the examiner or computer, which the participant must reproduce in the same order. The test has been widely applied across various populations, including children, adults, and clinical groups (Arce & McMullen, 2021; Castaldi et al., 2024; Pagulayan et al., 2006).

Despite its widespread use, the CBTT has been criticized for its lack of standardization in administration and scoring procedures (Claessen et al., 2015). Key characteristics—such as block layout, sequence length, presentation speed, and mode of administration (manual vs. computerized)—are often inconsistent across studies (Arce & McMullen, 2021). Moreover, there is ambiguity regarding what aspects of memory the test actually measures. Some researchers argue that the forward version of the CBTT primarily assesses short-term memory, while the backward version more accurately captures working memory (White et al., 2019). Others highlight the involvement of sequential processing, attention, and motor planning (Brunetti et al., 2014, 2018), further complicating interpretation.

Traditionally, most studies employing the Corsi Block-Tapping Test (CBTT) have focused on a single performance metric: Corsi span, defined as the longest sequence correctly recalled (Voyer et al., 2017). However, span scores alone may lack the sensitivity and reliability required to detect individual differences, particularly in developmental samples (Conway et al., 2005; White et al., 2018). To address these limitations, accuracy—operationalized as the number of correctly performed items—may be used as an alternative or complementary measure (Voyer et al., 2017). In contrast to the span, accuracy captures performance across the full range of trials and may provide more stable individual estimates. Despite its potential, another dimension—reaction time (RT)—remains notably underexplored in CBTT studies, even though it may yield valuable insights into processing speed, planning, and motor execution (Arce & McMullen, 2021).

These three CBTT performance metrics can reflect distinct cognitive mechanisms. While span and accuracy primarily reflect memory capacity, RT is more closely related to attentional control, executive planning, and motor coordination (Domingue et al., 2022; Fischer, 2001). For instance, Fischer (2001) found that performance improved with extended encoding and retention intervals and with fewer response options, implying that RT may be sensitive to task complexity and executive demands. Thus, a more nuanced analysis that integrates RT alongside span and accuracy could yield deeper insights into VSWM development and its relevance to academic achievement.

Despite a growing number of studies of VSWM, relatively few studies have concurrently examined span, accuracy, and RT in CBTT across school-aged children and adolescents, particularly with attention to gender differences. The aim of this study was two-fold. First, we aimed to examine age- and sex-related differences in CBTT performance across span, accuracy, and reaction time. Second, we aimed to evaluate the associations between CBTT performance metrics and math achievement, considering variations across grade levels and sex.

## Methods

### Sample

Data were collected from pupils in grades 5 to 11 in two regions in Russia, Nizhny Novgorod and Irkutsk **(***N* = 2,749; 53% girls). The mean age of participants was 13.89 years (*SD* = 2.08), with an age range of 10 to 18 years**.** *Table 1* presents the distribution of the sample by grade, along with the proportion of girls and the average age per group.

**Table 1:** Characteristics of Sample

Grade	*N* (%)	Proportion of girls (%)	Mean age (range)
5	539 (20%)	52%	11.3 (10 – 13)
6	481 (18%)	50%	12.3 (10 – 14)
7	413 (15%)	46%	13.3 (12 – 15)
8	212 (8%)	48%	14.6 (13 – 16)
9	160 (6%)	50%	15.3 (14 – 16)
10	849 (31%)	62%	16.2 (15 – 17)
11	95 (3%)	43%	17.3 (17– 18)

### Instruments and Variables

#### Corsi Block Tapping Test (CBTT)

The CBTT was adapted for online administration from the pen-and-paper version described by Pagulayan et al. (2006). To improve feasibility and internal validity for the school-aged sample, the number of trials per level was reduced based on results from pilot testing.

In the task, participants observe a sequence of glowing cubes and are required to reproduce the sequence by clicking the corresponding boxes. The task begins at Level 4 (sequences of 4 items), and includes two sequences per level across nine levels (total of 18 trials). If participants complete at least one correct sequence at a given level, they proceed to the next. The task ends when both sequences in a level are recalled incorrectly.

A visual instruction screen and one practice trial (3-item sequence) are provided, which can be repeated until the participant is comfortable. Each cube glows for 1 second, with a 1-second inter-stimulus interval. The software allows for breaks between trials and automatically records accuracy and reaction time (RT) for each trial.

Three performance indicators were derived:

- Accuracy: total number of correctly reproduced sequences.
- Corsi span: the maximum sequence length for which both sequences at that level were correctly recalled. For example, if a participant correctly completed both 5-item sequences but made an error in at least one of the 6-item sequences, their span would be 5.
- Average RT (for correct answers): mean reaction time across correctly recalled trials. RTs for incorrect responses were excluded, as they may reflect irrelevant or inconsistent processes (Whelan, 2008).

#### Math Achievement

As a proxy for academic performance, students’ school grades in mathematics (grades 5–6) or algebra and geometry (grades 7–11) were collected. An average math grade was calculated for each participant. School grades were used as they reflect students’ sustained performance across various classroom contexts and are considered ecologically valid indicators of academic functioning.

### Statistical Approach

We began by computing descriptive statistics for each CBTT performance metric: mean accuracy, mean reaction time (RT) for correct responses, and the median Corsi span. These metrics were examined for the overall sample and across grade levels. Participants who failed to correctly answer any trial (*N* = 108; 4% of the sample) were excluded from the analysis, under the assumption that such responses likely reflected inattentiveness or disengagement.

To examine age- and gender-related differences in CBTT performance, we conducted regression analyses. Because RT distributions were non-normal, we applied a natural log transformation to the average RT for correct responses. Linear regression models were used for accuracy and log-transformed RT, while ordinal regression was employed for the span variable. Each model included sex (coded as 1 = girl) and school grade as predictors, with grade treated as a categorical variable (reference category = grade 5). To explore whether sex differences varied across grades, interaction terms between sex and grade were subsequently included.

We next examined the relationship between CBTT performance and math achievement. Linear regression models predicted standardized math grades from CBTT accuracy and RT, along with grade and sex as covariates. To explore developmental and sex-specific patterns, we included interaction terms between grade, sex, and each CBTT metric. Accuracy and math grades were transformed to *Z*-scores prior to analysis for ease of interpretation.

The span measure was not included in models predicting math achievement for several reasons. First, as an ordinal measure with limited range, span scores may lack the sensitivity required to capture subtle individual differences in academic performance. Second, span scores are often less reliable than accuracy and RT, particularly in younger or lower-performing samples, where the early termination rule may limit variability. Finally, span and accuracy are often highly correlated, and including both may introduce redundancy in the models.

## Results

### Descriptive Statistics

We first computed the mean, median, and standard deviation for three CBTT indicators: accuracy (total number of correct trials), Corsi span, and average RT for correct responses. These statistics were calculated for the full sample and separately by school grade. Due to the small number of participants in grade 11, grades 10 and 11 were combined into a single group. *Table 2* presents the results.

**Table 2:** Descriptive Statistics for Accuracy, Span and Average RT for Correct Answers in CBTT, by Grade

Grade	Accuracy	Span	Average RT correct answers
	Mean (SD)	Median (IQR)	Mean (SD), msec
Total	4.90 (1.87)	5 (5–6)	4851.9 (2002.9)
5	4.18 (1.69)	5 (4–5)	5395.0 (1837.3)
6	4.37 (1.73)	5 (4–5)	4891.2 (1513.1)
7	4.82 (1.74)	5 (5–6)	4716.7 (1886.4)
8	5.19 (1.83)	5 (5–6)	4655.2 (2053.5)
9	5.14 (1.73)	5 (5–6)	4050.8 (956.4)
10–11	5.46 (1.90)	5 (5–6)	4771.3 (2367.5)

*Note: SD – standard deviation, IQR – interquartile range.*

The results show that accuracy increased steadily from grade 5 to 11, while the average RT for correct answers decreased over the same period. In contrast, the median span remained stable across grades, consistent with previous findings suggesting that span is a relatively insensitive measure of individual differences.

We also examined CBTT indicators by sex *(Table 3)*.

**Table 3:** Descriptive Statistics for Accuracy, Span and Average RT for Correct Answers, by Sex

Grade	Accuracy	Span	Average RT correct answers
	Mean (SD)	Median (IQR)	Mean (SD), msec
Boys	4.93 (1.82)	5 (5–6)	4678.3 (1834.6)
Girls	4.86 (1.90)	5 (5–6)	5004.7 (2129.1)

The results revealed that girls showed slightly lower accuracy and longer RT compared to boys.

**Figure 1:**
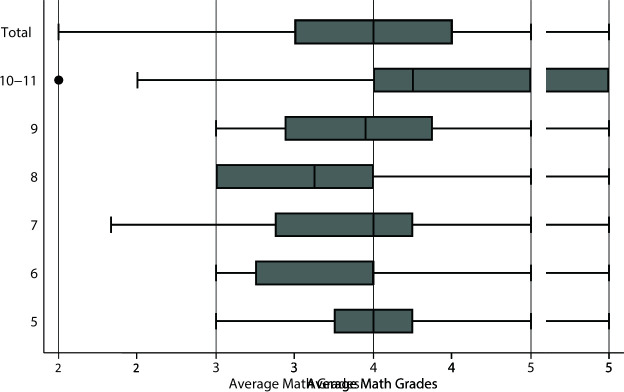
Distribution of Average Math Grades, by Grade

We also examined the distribution of average math grades, which ranged from 2 to 5 (M = 3.99, SD = 0.68). The distribution of these grades is illustrated in *Figure 1***.**

The results showed that the highest math grades were observed in grades 10-11, with a median value of 4.25. In contrast, the lowest math achievement was found in grade 8, where the median value was 3.625.

### Age and Sex Differences in CBTT: Regression Analysis Results

Linear regression models were estimated with accuracy (Z-scores) and log-transformed response time (RT) in correct answers as dependent variables. In Model 1, several predictors were included: gender (coded as girl = 1) and grade level (grade 5 served as reference group, with dummy variables created for the other grades). In Model 2, interaction terms between gender and each grade-level dummy variable were added to estimate whether gender difference varied across grades. Unstandardized regression coefficients (B) and standard errors are reported in *Table 4*, with accuracy as the dependent variable.

**Table 4:** Results of Regression Analysis for Accuracy (Z-scores) in CBTT

Variables	Model 1	Model 2
	B (s.e.)	B (s.e.)
Grade 6	.10 (.06)	.05 (.08)
Grade 7	.33*** (.06)	.26** (.09)
Grade 8	.54*** (.08)	.56*** (.12)
Grade 9	.51*** (.08)	.54*** (.12)
Grade 10-11	.69*** (.05)	.79*** (.08)
Girl	–.07* (.03)	–.05 (.08)
*Interaction*		
Grade6 * Girl		.09 (.12)
Grade7 * Girl		.16 (.12)
Grade8 * Girl		–.05 (.16)
Grade9* Girl		–.06 (.17)
Grade10-11* Girl		–.17 (.11)
*R*-squared	.076	.078

Note. ***p < .001, **p < .01, *p < .05

**Figure 2:**
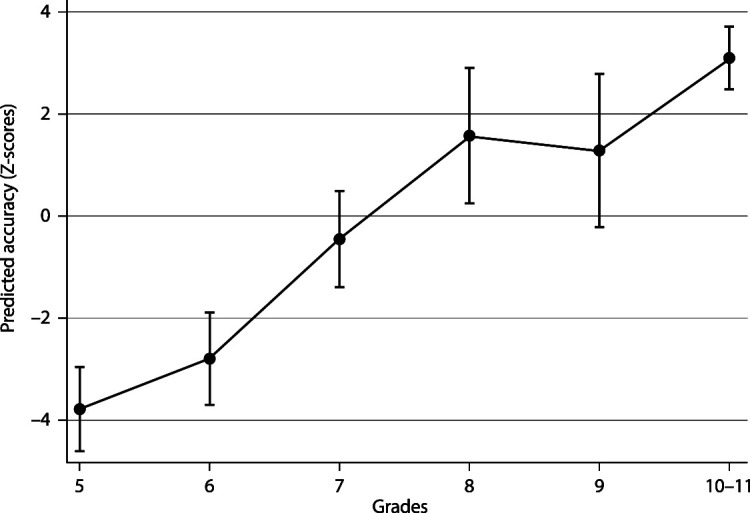
Predicted accuracy in CBTT (Z-scores, with 95% CI) from Grade 5 to Grade 10–11

The results from Model 1 indicated that accuracy increased almost linearly from grades 5 to 8, after which the increase slowed (*Figure 2*).

Sex differences in accuracy were minimal (standardized regression coefficient β = –.04), reflecting a very small effect size. Next, we included interactions between grade and sex to examine how sex differences in accuracy varied across years of education.

**Figure 3:**
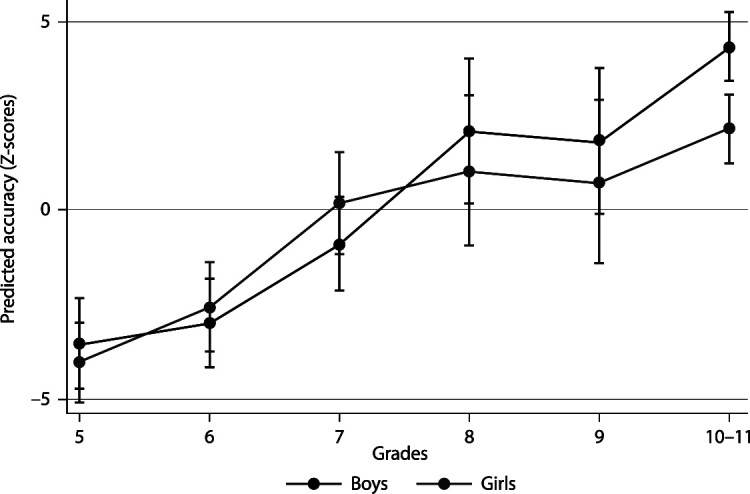
Predicted Accuracy in CBBT (Z-scores, with 95% CI), by Grade and Sex

In Model 2, while the overall fit did not improve, the inclusion of interaction terms provided a more nuanced understanding of sex differences. Specifically, the analysis revealed that, across grades 5 to 9, sex differences were not significant. However, for grades 10–11, boys demonstrated higher accuracy than girls (*Figure 3*).

Then, we ran regression analysis using the log-transformed average RT for correct answers as the dependent variable. Model 1 and Model 2 included the same predictors as in the accuracy analysis. Unstandardized regression coefficients (B) and standard errors are reported in *Table 5*.

**Table 5:** Results of Regression Analysis for RT for Correct Answers (Log-transformed) in CBTT

Variables	Model 1	Model 2
	B (s.e.)	B (s.e.)
Grade 6	–.09** (.02)	–.09*** (.03)
Grade 7	–.13*** (.02)	–.15*** (.03)
Grade 8	–.15*** (.02)	–.13*** (.04)
Grade 9	–.26*** (.02)	–.24*** (.04)
Grade 10–11	–.17*** (.02)	–.09*** (.03)
Girl	.06*** (.01)	.11*** (.03)
*Interaction*		
Grade6 * Girl		.01 (.04)
Grade7 * Girl		.04 (.04)
Grade8 * Girl		–.04 (.05)
Grade9* Girl		–.05 (.06)
Grade10–11* Girl		–.13*** (.03)
*R*–squared	.05	.06

***p < .001, **p < .01, *p < .05

**Figure 4:**
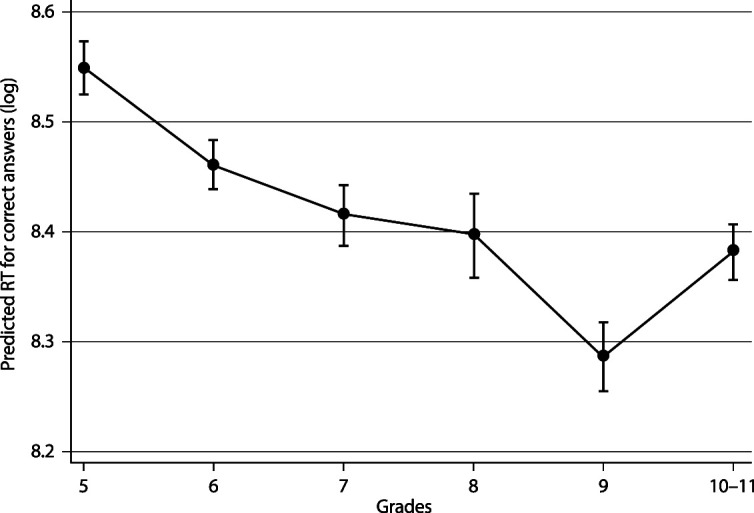
Predicted RT for Correct Answers (Log-transformed), by Grade

Model 1 results showed a general decrease in RT across grades, with significant reductions through grade 9, followed by a slight increase in later grades (*Figure 4*).

**Figure 5:**
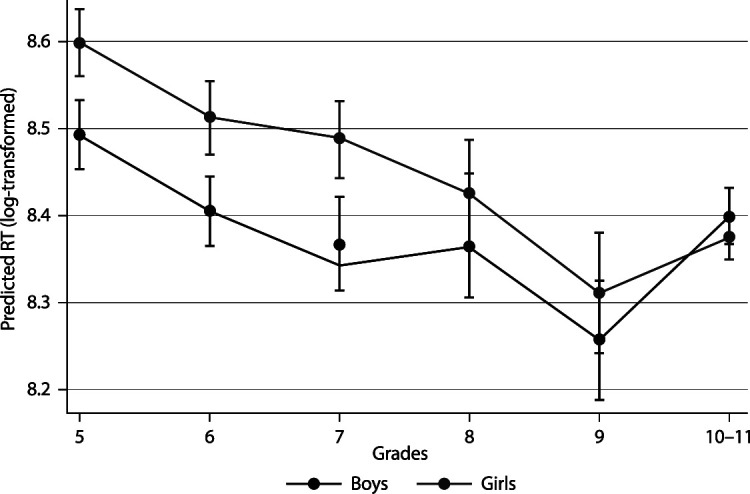
Predicted Average RT for Correct Answers (Log-transformed), by Sex and Grade

Sex differences in RT were significant, with girls being slower on average than boys (standardized regression coefficient β = .09). However, the interaction analysis (Model 2) revealed that the only significant interaction was between grade 10–11 and sex, indicating that boys were faster than girls in earlier grades, while this trend reversed in the 10–11 grades, although the difference was not significant **(***Figure 5***).**

Due to the restricted range of values for the span indicator, we applied an ordinal regression to the span measure, excluding cases where the span was 0 (*i.e*., only 1 correct answer). *Table 6* presents the results in terms of logit coefficients (B), with standard errors in parentheses.

**Table 6:** Results of Ordinal Regression Analysis for Corsi Span

Variables	Model 1	Model 2
	B (s.e.)	B (s.e.)
Grade 6	.16 (.12)	.10 (.18)
Grade 7	.60*** (.13)	.34 (.18)
Grade 8	.57*** (.13)	.93*** (.22)
Grade 9	.92*** (.16)	.88*** (.24)
Grade 10-11	.88*** (.17)	1.42*** (.16)
Girl	–.17** (.07)	–.18 (.17)
*Interaction*		
Grade6 * Girl		.14 (.24)
Grade7 * Girl		.51* (.25)
Grade8 * Girl		–.003 (.31)
Grade9* Girl		.002 (.34)
Grade10-11* Girl		–.26 (.21)
Threshold 1 (from 4 to 5)	–.88 (.09)	–.89 (.13)
Threshold 2 (from 5 to 6)	1.17 (.10)	1.17 (.13)
Threshold 3 (from 6 to 7)	2.99 (.11)	2.99 (.14)
Threshold 4 (from 7 to 8)	5.12 (.20)	5.13 (.22)
R-squared	.03	.03

Note. ***p < .001, **p < .01, *p < .05

**Figure 6:**
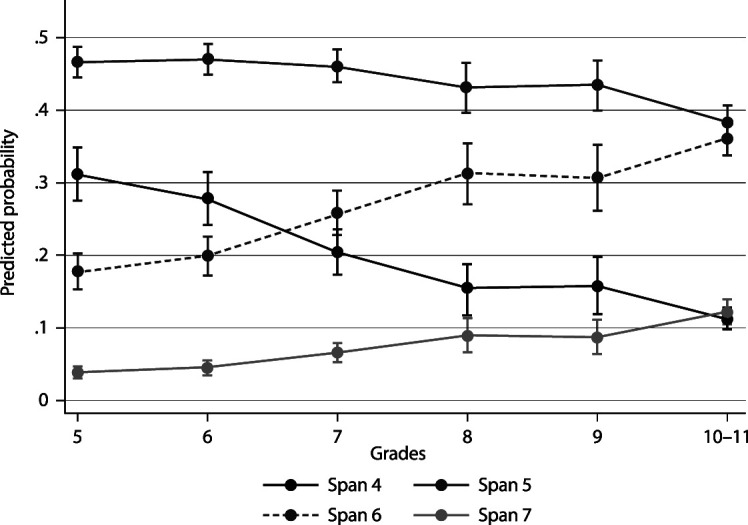
Probability of Different Values of Span in CBTT, by Grade

The results of Model 1 for span revealed that girls had a lower probability of achieving higher span values. The probability of achieving higher span increased across years of education. Post-hoc estimates indicated that the probability of a span value of 5 was highest and remained stable across grades. The probability of span 4 decreased, while span 6 became more likely over time. The probability of span 7 remained low, increasing only in grades 10–11 (*Figure 6*).

**Figure 7:**
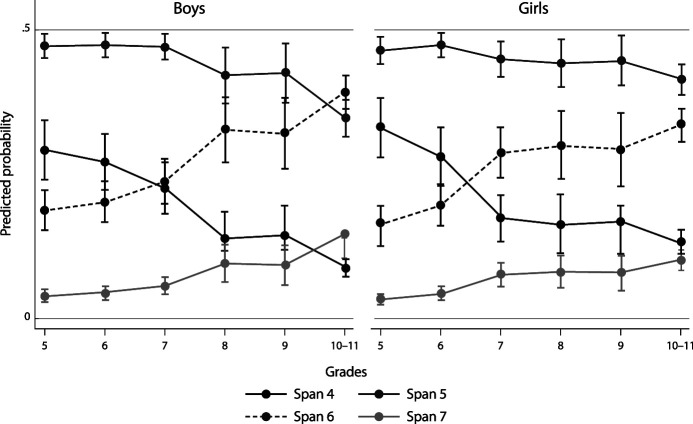
Probability of Different Values of Span in CBTT, by Grade and Sex

The analysis of sex differences in span revealed a stable pattern across grades. In Model 2, only one interaction term was significant, indicating that sex differences in span were generally consistent. However, post-hoc estimates revealed some differences between boys and girls. In grades 10–11, the probability of span 7 became higher than span 4 for boys, while this was not the case for girls. Additionally, in grades 10–11, girls had a higher probability of achieving span 5, while boys showed similar probabilities for spans 5 and 6 (*Figure 7*).

### CBTT and Math Achievement: Results of Regression Analysis

We examined the relationship between two CBTT indicators—accuracy (*Z*-scores) and log-transformed average RT for correct answers—and students’ math achievement, measured by average school grades in mathematics (also standardized as *Z*-scores). The Corsi span was excluded from this analysis due to low variability and a lack of developmental change across grades.

To examine whether the relationships between CBTT performance and math achievement varied by grade level or sex, we estimated a series of linear regression models. The dependent variable was the average math grade (standardized as a *Z*-score), and the key predictors were CBTT accuracy (standardized) and log-transformed reaction time (RT) for correct responses.

Model 1 included the two CBTT indicators along with grade-level dummies (grade 5 as the reference category) and a binary variable for sex (girl = 1). In Models 2 and 3, we added interaction terms between each CBTT variable and grade level to test whether the associations with math achievement differed across grades. In Models 4 and 5, we included interaction terms between each CBTT variable and sex to assess whether the strength of associations varied for boys and girls.

Results of the five regression models, including unstandardized coefficients (B) and standard errors, are presented in *Table 7*.

**Table 7:** Results of Regression Analysis for Average Math Grades (in Z-scores)

Variables	Model 1	Model 2	Model 3	Model 4	Model 5
	B (s.e.)	B (s.e.)	B (s.e.)	B (s.e.)	B (s.e.)
Accuracy CBTT (Z-scores)	.14*** (.02)	.24*** (.05)	.14*** (.02)	.15*** (.03)	.14*** (.02)
RT CBTT (log)	–.21** (.06)	–.19** (.06)	–.25 (.15)	–.21** (.06)	–.16 (.09)
Grade 6	–.13* (.06)	–.15* (.06)	–.96 (1.94)	–.13* (.06)	–.13* (.06)
Grade 7	–.30*** (.06)	–.33*** (.07)	–1.84 (1.90)	–.30*** (.06)	–.30*** (.06)
Grade 8	–.59*** (.08)	–.64*** (.08)	–1.20 (2.35)	–.59*** (.08)	–.59*** (.08)
Grade 9	–.26** (.09)	–.31** (.09)	1.23 (3.27)	–.26** (.09)	–.26** (.09)
Grade 10-11	.11 (.06)	.13* (.06)	.05 (1.49)	.11 (.06)	.10 (.06)
Girl	.37*** (.04)	.35*** (.04)	.36*** (.04)	.37*** (.04)	1.15 (1.04)
*Interaction*					
Grade6* accuracy		–.03 (.07)			
Grade7* accuracy		.01 (.07)			
Grade8* accuracy		–.01 (.08)			
Grade9* accuracy		–.004 (.09)			
Grade10-11* accuracy		–.29*** (.06)			
Grade6 * RT			.10 (.23)		
Grade7 * RT			.18 (.22)		
Grade8 * RT			.07 (.28)		
Grade9* RT			–.18 (.39)		
Grade10-11* RT			.01 (.18)		
Girl*accuracy				–.02 (.04)	
Girl*RT					–.09 (.13)
R-squared	.10	.11	.10	.10	.10

Note. ***p<.001, **p<.01, *p<.05

The results demonstrated that both CBTT accuracy and RT were significantly associated with math performance: higher accuracy predicted better math grades, while slower RT (i.e., higher log RT) was associated with lower performance. Additionally, math grades generally declined with increasing grade level, except for grades 9–11, where the trend stabilized.

**Figure 8:**
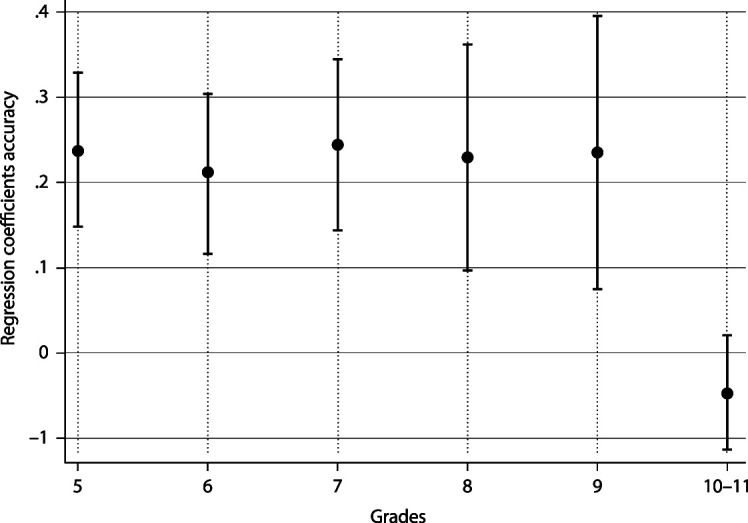
Regression coefficients (with 95% CIs) for CBTT accuracy, by grade

Model 2, which included interactions between grade and CBTT accuracy, showed that the positive relationship between accuracy and math achievement was consistent from grades 5 through 9. However, in grades 10–11, the association weakened and became non-significant. This trend is illustrated in *Figure 8*.

In Model 3, none of the interaction terms between RT and grade were significant, indicating that the negative association between RT and math performance was stable across all grade levels.

Finally, Models 4 and 5 tested whether the strength of these associations varied by sex. Interactions between sex and both CBTT accuracy and RT were not significant, suggesting that the relationships between CBTT performance and math achievement were similar for boys and girls.

## Discussion

This study had two aims. First, we examined age- and sex-related differences in VSWM, as measured by the CBTT, using three indicators: accuracy, span and average RT for correct answers. Second, we estimated how accuracy and RT in CBTT relate to math achievement across school grades and sexes.

Our findings highlight the importance of using multiple CBTT performance metrics to better understand the developmental trajectory of visuospatial working memory (VSWM). While traditional reliance on the Corsi span has been widespread, it may overlook meaningful individual differences (White et al., 2018; Claessen et al., 2015). In contrast, accuracy and reaction time (RT) provide complementary insights: accuracy reflects the fidelity of memory encoding and attentional maintenance, while RT offers information about cognitive efficiency, executive planning, and motor coordination (Domingue et al., 2022; Fischer, 2001).

### Age-Related Differences

Following Tikhomirova et al. (2020a), we used school grade as an indicator of years of education rather than chronological age, given that educational experience often better explains cognitive development in school-aged populations. Consistent with previous research showing non-linear developmental trajectories in CBTT performance (e.g., Tikhomirova et al., 2020a), our results also revealed a non-monotonic pattern across grades. From grades 5 to 8, accuracy increased while RT for correct answers decreased, suggesting concurrent improvements in memory precision and processing speed. Between grades 8 and 9, accuracy plateaued while RT continued to decline, possibly reflecting increasing automatization of task performance. Interestingly, after grade 9, both accuracy and RT increased again, indicating a potentially distinct developmental phase characterized by different cognitive or strategic processes.

These findings may reflect the involvement of different mechanisms underlying CBTT performance. Early improvements may be primarily driven by maturation of core cognitive functions, while later changes might involve increased use of strategies, greater engagement with complex tasks, or shifts in educational demands. These findings suggest that the divergence in developmental trajectories of RT and accuracy in CBTT may reflect the maturation of distinct underlying neural systems. While RT improvements may correspond to increasing processing speed due to myelination and pruning (Kwon et al., 2020), improvements in accuracy may reflect the strengthening and stabilization of memory traces and attentional control (Spencer, 2020). Thus, our observed patterns—initial parallel improvement in both metrics, followed by decoupling in later grades—may represent developmental shifts in the dominant cognitive mechanisms supporting task performance. This interpretation aligns with recent findings in numerical cognition, where similar dissociations in developmental trends in RT and accuracy have been documented (Malykh et al., 2021). Future longitudinal and neurophysiological studies are needed to verify whether such patterns in CBTT are indeed driven by distinct neurocognitive systems with different developmental timelines.

Previously, it was shown that VSWM undergoes two periods of stabilization: one during late childhood and another during late adolescence (Ahmed et al., 2022). However, our findings further suggest that development may continue even into late adolescence (ages 15–17), as accuracy in CBTT significantly increased from grades 9 to 10–11.

At the same time, these results must be interpreted with caution. In Russia, compulsory education ends after grade 9, and many students transition to vocational tracks. Those who remain in general secondary education (grades 10–11) tend to be a selective group, often characterized by higher academic achievement, socioeconomic status (SES), and parental education levels (Khavenson & Chirkina, 2019). Therefore, the observed increases in CBTT performance in grades 10–11 may partly reflect selection effects rather than universal developmental gains. This highlights the importance of considering contextual factors such as educational system structure and population composition when interpreting developmental trends.

### Developmental Dynamics of VSWM-Math Link

Consistent with previous research (e.g., Vieira et al., 2021; Allen & Dowker, 2022), we found that CBTT accuracy was positively associated with math performance, supporting the view that VSWM plays a significant role in academic learning, particularly in mathematics.

In line with earlier findings (Tikhomirova et al., 2020b), we observed that the strength of the association between CBTT accuracy and math achievement was not uniform across grades. The association was robust from grades 5 to 9 but significantly weakened in grades 10–11. This pattern may reflect a developmental shift in the cognitive strategies used to solve math problems. Younger students are more likely to rely on visuospatial strategies and mental representations (Allen et al., 2019), whereas older students may increasingly depend on abstract reasoning, verbal strategies, or metacognitive skills that reduce reliance on VSWM (Pekrun et al., 2007; Tachie, 2019; Winsler & Naglieri, 2003).

Reaction time was negatively associated with math achievement across all grades. This stable relationship suggests that faster and more efficient processing during visuospatial tasks supports better mathematical performance, potentially by reducing cognitive load and freeing up resources for problem-solving. Notably, while accuracy showed developmental variability in its predictive power, RT’s effect remained constant, suggesting that these metrics tap into distinct yet relevant aspects of cognition.

### Sex Differences

Contrary to some prior research suggesting sex differences in the strength of WM–math associations (Van de Weijer-Bergsma et al., 2021), we found no sex-based moderation in the relationship between CBTT performance and math grades. This may be partly due to the diminishing sex difference in VSWM by mid-to-late adolescence (Tikhomirova et al., 2020a). However, we found small sex differences in late adolescence in trends for both accuracy and RT in CBTT. Particularly, results revealed that from grades 9 to 10–11, girls had a slightly lower increase in accuracy and in RT than boys. This indicated that despite less effective memory encoding and attentional maintenance in girls, they became more effective in terms of speed of processing, planning, and motor coordination. These results are in line with previous studies of sex differences in processing speed during adolescence, which demonstrated the advantage of girls in processing speed or the absence of significant sex differences (e.g., Camarata & Woodcock, 2006; Roivainen et al., 2021).

## Conclusion

In summary, this study reveals that both accuracy and RT in the CBTT provide distinct and valuable insights into the development of visuospatial working memory and its relationship with math achievement across adolescence. Our findings underscore the importance of using multiple performance metrics, highlight nonlinear and stage-specific patterns of cognitive growth, and show that while accuracy becomes less predictive of math performance in late adolescence, RT remains a stable predictor across all grades. The results suggest that changes in VSWM over time may reflect the maturation of different neurocognitive systems, with developmental, strategic, and contextual factors all playing a role. Importantly, these trajectories are influenced by educational structures, with selection effects in upper grades warranting cautious interpretation. Overall, this study contributes to a more nuanced understanding of VSWM development and its academic correlates, while pointing to the need for longitudinal, multi-method research to disentangle individual and contextual influences.

## Limitations and Future Directions

Several limitations should be acknowledged. First, the cross-sectional design precludes causal inferences about the developmental trajectories observed. Longitudinal studies are needed to verify whether the decline in VSWM’s predictive power is truly age-related or influenced by curriculum changes.

Second, we used aggregated indicators of CBTT (accuracy or RT). Recent studies have demonstrated that, for better understanding of students’ ability and assessing complex relationships between accuracy and RT, item-level analysis should be considered (e.g., mixed-effects or IRT models) (Brauer & Curtin, 2018; Lo & Andrews, 2015; Molenaar et al., 2015).

Third, in estimating the association between VSWM and math, we did not control for other cognitive functions that could explain this association, such as intelligence or processing speed (Tikhomirova et al., 2020b).

Finally, math achievement was measured using school grades, which, while ecologically valid, may not purely reflect mathematical ability. Grades can be influenced by a range of non-cognitive factors, including motivation, classroom behavior, teacher expectations, and socio-emotional skills, potentially confounding the observed associations with cognitive variables. Using standardized or task-specific assessments in future research may provide a more accurate estimation of students’ math proficiency.

Additionally, future studies could investigate whether different types of math tasks (e.g., arithmetic vs. geometry, procedural vs. conceptual) draw differentially on VSWM resources, and whether contextual factors, such as teaching methods or cognitive training, influence the observed associations.
